# DSC-LLM: Driving Scene Context Representation-Based Trajectory Prediction Framework with Risk Factor Reasoning Using LLMs †

**DOI:** 10.3390/s25237112

**Published:** 2025-11-21

**Authors:** Sunghun Kim, Joobin Jin, Seokjun Hong, Dongho Ka, Hakjae Kim, Byeongjoon Noh

**Affiliations:** 1 Department of AI and Big Data, Soonchunhyang University, 22 Soonchunhyang-ro, Asan 31538, Republic of Korea; ksh0816@sch.ac.kr; 2 Department of Future Convergence Technology, Soonchunhyang University, 22 Soonchunhyang-ro, Asan 31538, Republic of Korea; jjb0821@sch.ac.kr (J.J.); hongsj@sch.ac.kr (S.H.); 3 ITS Team, Nota AI Inc., 521 Teheran-ro, Gangnam-gu, Seoul 06164, Republic of Korea; dongho.ka@nota.ai; 4 R&D Department, Class Act Co., Ltd., 123, Gasan Digital 2-ro, Geumcheon-gu, Seoul 08505, Republic of Korea

**Keywords:** trajectory prediction, driving scene context, risk-aware reasoning, multimodal LLM, autonomous driving systems

## Abstract

Autonomous driving in dense urban environments requires accurate trajectory forecasting supported by interpretable contextual evidence. This study presents a multimodal framework that performs driving scene context (DSC)-aware trajectory prediction while providing risk-aware explanations to reveal the contextual cues behind predicted motion. The framework integrates temporal object states—trajectories, velocities, yaw angles, and motion status—with semantic information from forward-facing camera imagery, and is composed of four modules: object behavioral feature extraction, scene context extraction, DSC-augmented trajectory prediction, and risk-aware reasoning using a multimodal large language model (MLLM). Experiments on the Rank2Tell dataset demonstrate the feasibility and applicability of the proposed approach, achieving an ADE of 10.972, an FDE of 13.701, and an RMSE of 8.782. Additional qualitative evaluation shows that DeepSeek-R1-Distill-Qwen-7B generates the most coherent and contextually aligned explanations among the tested models. These findings indicate that combining DSC-aware prediction with interpretable reasoning provides a practical and transparent solution for autonomous driving in complex urban environments.

## 1. Introduction

Recent advancements in autonomous driving have demonstrated remarkable performance across various tasks, including perception, planning, and control. However, achieving reliable operation in dense and unpredictable urban environments remains an open challenge, primarily due to the complex and dynamic nature of city traffic [1,2,3,4,5]. Real-world urban scenarios frequently involve irregular events such as pedestrians crossing roads outside of designated crosswalks or illegally parked cars obstructing traffic lanes. These uncertain and potentially dangerous situations demand real-time situational awareness and prompt, appropriate decision-making by autonomous systems [6,7,8].

A key capability for navigating such complexities is the accurate prediction of surrounding agents’ future movements, especially those of pedestrians and nearby vehicles [9,10]. While many trajectory prediction techniques have been proposed, most rely primarily on historical position data or past motion patterns. Such methods often fall short in environments where contextual cues exert a significant influence on object behavior [11,12,13,14,15]. To address this gap, integrating visual context into trajectory forecasting is essential for improving prediction accuracy and robustness in dense, interactive environments.

Beyond predicting the movements of surrounding agents, it is equally critical for autonomous driving systems to provide interpretable justifications for their decision-making processes in safety-critical situations. Conventional black-box models often fail to articulate the rationale behind specific control actions, making it challenging for operators, auditors, or passengers to evaluate the appropriateness of system behavior under complex and dynamic conditions. To ensure operational transparency and trust, autonomous vehicles should generate human-understandable explanations that explicitly link perceived environmental cues to subsequent driving decisions. This interpretability must include the identification of high-risk objects or hazardous events in the surrounding environment, the assessment of their potential impact on driving safety and the recommendation of appropriate control strategies to mitigate these risks [16].

Recent developments in large language models (LLMs), particularly multimodal or vision-language models (VLMs), have shown promise in bridging this gap. These models can not only interpret complex visual scenes but also perform higher-level reasoning to produce coherent descriptions in natural language that highlight potential risks and their implications for driving decisions [17,18,19,20,21]. Nevertheless, most existing works treat trajectory prediction and risk-aware explanation as disjoint tasks, lacking an integrated approach that provides both predictive and explanatory outputs for comprehensive driving intelligence.

In this study, we propose a novel multimodal framework that jointly performs driving scene context (DSC)-aware trajectory prediction and risk-aware decision reasoning by integrating video footage, object behavioral features, and textual scene descriptions. The system utilizes temporal object state information—such as trajectories, velocities, yaw angles, and dynamic/static status—together with semantic scene context extracted from front-facing camera imagery. The proposed framework consists of four tightly connected modules. First, the Object Behavioral Feature Extraction module encodes temporal dynamics through a temporal multimodal embedding (TME) layer implemented with recurrent and Transformer-based encoders. Second, the Scene Context Feature Extraction module processes raw video streams and corresponding textual descriptions using vision and text encoders, followed by cross-attention-based feature fusion. Third, the Driving Scene-Aware Trajectory Prediction module integrates object features and contextual cues, generating future motion trajectories through Transformer-based encoders and decoders guided by dynamic/static queries. Finally, the Risk-Aware Decision Reasoning module employs a multimodal LLM to identify the most hazardous entities, explain their risk levels in natural language, and recommend appropriate driving responses.

Rather than relying on additional large-scale language model training, this study employs a pretrained multimodal LLM to evaluate the feasibility and explanatory capability of the proposed reasoning mechanism using structured scene representations. By combining trajectory forecasting with explanation generation, the proposed framework offers both quantitative motion predictions and qualitative, human-understandable reasoning within a unified architecture, underscoring the importance of addressing these two tasks in a coherent manner rather than treating them independently. In fact, this study extends our previous work [21] through substantial architectural improvements, refined experiments, and the inclusion of multi-seed statistical evaluation as well as additional qualitative analyses for a more thorough examination of system behavior. Importantly, the framework operates solely on camera input and lightweight object state features, eliminating the need for costly sensing equipment such as LiDAR while still achieving strong predictive accuracy and maintaining explanatory clarity, thereby offering a scalable and cost-effective approach for autonomous driving in complex urban environments. We validated the feasibility and applicability of the proposed framework by implementing it and applying it to the Rank2Tell benchmark [22] for comprehensive evaluation, thereby confirming its practical potential under diverse driving scenarios.

In addition, the proposed DSC-based LLM reasoning can be utilized in both the prediction phase and the post-hoc analysis phase of real autonomous driving. During operation, it supports safer trajectory planning by incorporating contextual cues into motion forecasting and provides explicit evidence of potential hazards when the risk of an accident increases. After driving, it can reconstruct the rationale behind specific behaviors or decisions in an interpretable manner, serving as an essential basis for system validation, auditing, accident investigation, and policy-level refinement.

## 2. Related Work

### 2.1. Trajectory Prediction

Forecasting the future motion of surrounding agents, such as vehicles and pedestrians, is a fundamental component of the decision-making pipeline of autonomous driving systems. This task becomes particularly critical in urban environments, where road users exhibit highly dynamic and interactive behaviors. In this work, we consider all observable agents within the forward-facing camera view of the ego vehicle as prediction targets.

Traditionally, trajectory prediction has been addressed as a sequential modeling problem, where future positions are inferred based on observed historical trajectories. Early models often leveraged recurrent neural networks, particularly bidirectional long short-term memory (Bi-LSTM) architectures, to capture temporal dependencies in motion histories of agents [23,24,25,26,27]. These models demonstrated effective short-term prediction capabilities but were limited in handling complex agent interactions.

To model social dynamics and spatial dependencies among multiple agents, subsequent approaches incorporated graph neural networks (GNNs), which enabled structured modeling of inter-agent relationships in shared environments [28,29,30]. This class of methods has proved especially useful in crowded scenes, where interaction-aware reasoning is essential.

More recently, Transformer-based models have gained prominence due to their ability to capture long-range temporal patterns and global dependencies more efficiently than traditional recurrent models [31,32]. Notably, models such as the long-term time series forecasting normalization linear (LTSF-NLinear) have exhibited strong performance in long-horizon forecasting, making them well-suited for trajectory prediction tasks requiring anticipation over extended time windows [33].

In addition to motion history, the incorporation of high-level semantic context has been shown to further improve prediction performance. For instance, several methods utilize lane topology or HD map information obtained from LiDAR-based sources. These inputs are used to guide predictions that follow plausible driving paths and road constraints [34,35,36].

Beyond pure trajectory forecasting, recent efforts have explored the prediction of social behaviors and underlying intentions of agents. These approaches aim to capture the rationale behind motion patterns rather than merely spatial coordinates. In this vein, multimodal frameworks integrating visual features and language models have emerged. For example, LLM-based models have been employed to generate interpretable descriptions of object behaviors and scene dynamics, enhancing transparency and flexibility in prediction pipelines [37,38]. A notable instance is LG-Traj [39], which combines Transformer-based encoders with LLMs to effectively model pedestrian intentions and contextual cues, enabling more socially compliant forecasting.

Building upon these previous advances, this study proposes a trajectory prediction framework that effectively integrates the temporal motion dynamics of surrounding agents with contextual visual cues extracted from the driving environment. By jointly combining behavior sequences and environmental semantics, the proposed approach enables accurate and context-aware forecasting of future trajectories, particularly suitable for complex and dynamic urban driving scenarios.

### 2.2. Scene Understanding in Autonomous Driving Systems

With the growing sophistication of LLMs, recent studies have increasingly explored their integration into autonomous driving systems to improve situational awareness and model interpretability. A key focus in this line of research is enabling vehicles to articulate their decision-making logic in natural language, thereby enhancing transparency and user trust [40,41]. In contrast to conventional black-box models, LLM-based approaches aim to provide human-understandable explanations that clarify system behavior under dynamic and uncertain driving conditions [42].

One promising direction involves converting structured data such as object velocity, heading angle, and spatial position, into natural language expressions that align with the latent space of language models. This data-to-text translation process allows the model to represent its internal decision rationale more explicitly [43]. For example, synthetic driving datasets have been supplemented with descriptive annotations or driving-specific Q&A templates to guide LLM training for scene interpretation tasks [44,45].

To model more effectively the complex interplay between structured vector inputs and textual outputs, hybrid architectures combining Transformer encoders with vector representations have been developed. VectorFormer [46] is one such approach that uses Transformer layers to bridge numeric and linguistic modalities, enabling more nuanced understanding of agent interactions within a scene. Additionally, parameter-efficient tuning techniques such as Low-Rank Adaptation (LoRA) have facilitated the fine-tuning of large models while maintaining computational efficiency, making real-time applications more feasible [47].

Multimodal frameworks that combine object-level numerical features with scene-level semantic cues have also emerged, demonstrating improved capabilities in generating interpretable, task-relevant outputs. These models tokenize both structured and unstructured inputs to provide richer representations of driving scenes and generate well-informed recommendations for navigation or risk mitigation [48,49,50,51]. Collectively, these advancements represent a shift from accuracy-centric models toward systems that incorporate interpretability and context-aware reasoning as core functionalities in autonomous driving. However, despite their promise, most existing methods treat trajectory prediction and natural language reasoning as separate components. Integrated frameworks that can simultaneously predict motion paths and explain risk factors in real time remain underexplored.

To address this gap, we propose a unified framework that jointly performs trajectory prediction and interpretable risk-aware reasoning. By combining dynamic behavioral features with visual context and integrating LLM-based reasoning, the proposed system enhances both predictive accuracy and transparency in complex urban driving scenarios.

## 3. Methodology

**Figure 1:**
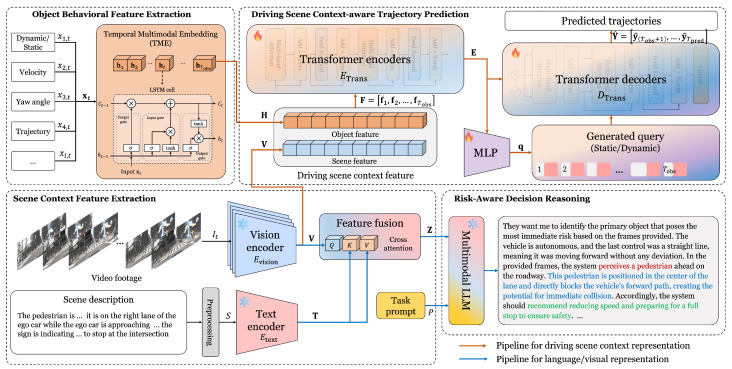
Overall architecture of the proposed framework. Colored text in the example MLLM output indicates semantic components used in risk-aware reasoning: red highlights the identified risky object, blue denotes the explanation for the risk, and green represents the recommended driving action.

In this section, we describe the proposed framework in Figure 1 that integrates driving scene context with MLLM-based reasoning to predict object trajectories and provide explainable risk-aware decisions. The proposed framework consists of four main modules: (1) object behavior feature extraction (2) scene context feature extraction (3) driving scene context-augmented trajectory prediction (4) risk-aware decision reasoning.

Compared to our previous version [21], we redesigned the overall modeling pipeline and expanded the experimental setup to achieve more stable and reliable performance across diverse driving scenarios. In particular, we refined the trajectory prediction component by modifying the Transformer encoder–decoder architecture, incorporating an MLP-based query generation mechanism for the decoder, and making a minor adjustment to the feature-fusion strategy to more effectively align behavioral and scene context representations. In addition, the multimodal LLM module employs an enhanced chain-of-thought (CoT) prompting strategy tailored for risk-aware reasoning, resulting in more structured and context-grounded explanations with improved consistency.

### 3.1. Object Behavior Feature Extraction

The primary objective of the object behavior feature extraction module is to construct temporally aligned representations that capture the motion dynamics of surrounding objects during the observation period $t=\{1,\ldots ,T_{\mathrm{obs}}\}$. Each motion state vector $x_{t}$ comprises *k* features, formally expressed as

(1) $$x_{t}=\left[x_{1,t},x_{2,t},\ldots ,x_{k,t}\right]$$

where the elements include trajectory coordinates, velocities, yaw angles, and dynamic/static indicators. These motion attributes together describe the object’s spatial evolution and behavioral intention over time.

Trajectories provide a historical record of position changes, offering spatial information over time. Velocities are computed as the displacement between consecutive positions, capturing the magnitude and direction of motion. Yaw angles represent the heading direction of each object, reflecting its orientation relative to the road or other agents. Dynamic states indicate whether an object is currently moving or stationary, providing coarse-grained cues about behavioral intention. By concatenating these features at each time step, a temporally ordered sequence of behavioral states is obtained.

To model the temporal evolution of these behaviors, we employ the TME, a recurrent architecture designed to efficiently encode long-range sequential dependencies. The TME processes the motion feature sequence and returns contextualized hidden representations for all observation steps:

(2) $$H=\mathrm{TME}\left(x_{1},\ldots ,x_{T_{\mathrm{obs}}}\right)=\left[h_{1}=\mathrm{LSTM}\left(x_{1}\right),\ldots ,h_{T_{\mathrm{obs}}}=\mathrm{LSTM}\left(x_{T_{\mathrm{obs}}}\right)\right]$$

Unlike conventional LSTM models, which may output only a final hidden state, the TME provides a hidden representation at every time step, enabling the model to preserve both instantaneous motion cues and accumulated temporal context. This fine-grained temporal modeling is essential for accurately capturing how behavioral patterns evolve over time.

The resulting sequence H serves as the behavioral embedding for downstream modules, including trajectory prediction with multimodal context fusion. By transforming raw motion signals into a temporally enriched representation, this module establishes the foundation for robust and context-aware trajectory prediction in complex driving environments.

### 3.2. Scene Context Feature Extraction

This module extracts and integrates global visual context from the driving environment to support both trajectory prediction and risk-aware decision reasoning. As object trajectories are often influenced by surrounding road layouts, dynamic agents, and static obstacles, modeling such visual context is critical for interpreting object behavior in real-world scenarios. To capture the visual semantics at each observation time step *t*, the forward-facing RGB image $I_{t}$ is processed by a contrastive language-image pre-training (CLIP) model [52]-based vision encoder $E_{\mathrm{vision}}$, producing a visual embedding.

(3) $$v_{t}=E_{\mathrm{vision}}\left(I_{t}\right),\;V=\left[v_{1},\ldots ,v_{T_{\mathrm{obs}}}\right]$$

Here, V denotes the temporally ordered sequence of visual embeddings extracted from consecutive video frames, providing a time-aligned representation of the evolving scene context. This sequence allows the model to associate motion patterns with the corresponding visual observations at each time step. The encoder transforms each image into high-dimensional feature maps that encode spatial and semantic information about the environment, such as road boundaries, crosswalks, parked vehicles, and pedestrian zones. These visual features are then temporally aligned and fused with motion embeddings derived from the object behavior feature extraction module, resulting in a joint representation referred to as the “driving scene context (DSC).” This fusion allows the model to interpret motion patterns within their environmental context, improving the accuracy of trajectory predictions.

In addition to visual encoding, the module incorporates linguistic information by processing natural language descriptions associated with the scene. Given a predefined scene description *S*, a pretrained language encoder $E_{\mathrm{text}}$ produces a semantic embedding, $T=E_{\mathrm{text}}\left(S\right)$.

To integrate linguistic and visual cues, the visual embedding sequence V is used as queries (Q), while the text embedding T serves as keys (K) and values (V) in a cross-attention mechanism. The resulting multimodal embedding is computed as

(4) $$Z=\mathrm{CrossAttn}\left(Q=V,\;K=T,\;V=T\right)=\mathrm{softmax}\;\left(\frac{QK^{⊤}}{\sqrt{d}}\right)V$$

where *d* denotes the dimensionality of the query and key embeddings used for scaling in the attention computation.

This attention-based fusion enables the model to highlight image regions that are most relevant to the given textual context, effectively bridging visual perception with language-guided reasoning. The fused multimodal representation Z is then forwarded to the risk-aware decision reasoning module, while the visual embedding V is passed to the driving scene context-aware trajectory prediction module for motion–context fusion. Through this alignment, the module achieves a comprehensive understanding of the driving environment, enabling nuanced reasoning about potential risks and generating context-sensitive driving responses.

### 3.3. Driving Scene Context-Augmented Trajectory Prediction

This module is designed to generate accurate and context-aware future trajectory predictions by leveraging a fused representation of temporal motion features and visual scene information. It adopts a transformer-based encoder-decoder architecture that enables the integration of both local temporal dynamics and global spatial semantics. Unlike conventional autoregressive approaches that estimate positions step-by-step, this module follows a non-autoregressive strategy to produce all future positions simultaneously. This design significantly reduces inference latency and mitigates the compounding errors commonly encountered in sequential prediction schemes [53].

The input to the encoder consists of temporally aligned feature sequences for each object. These sequences are obtained by fusing behavior representations—derived from motion history and dynamic states—with scene-level visual context features extracted from the scene context feature extraction module. Formally, let the fused input sequence be

(5) $$F=\left[f_{1},\ldots ,f_{T_{\mathrm{obs}}}\right],f_{t}=\left[h_{t};v_{t}\right]$$

where each $f_{t}$ represents a concatenation of temporally encoded behavioral features $h_{t}$ and visual scene features $v_{t}$.

The transformer encoder processes this fused representation. Specifically, the encoder $E_{\mathrm{Trans}}$ produces contextualized embeddings as

(6) $$E=E_{\mathrm{Trans}}\left(F\right).$$

A multi-layer perceptron (MLP) then generates decoder queries, q=MLP(E) where q provides coarse motion cues that guide the decoder during trajectory generation.

The decoder subsequently applies multi-head attention over the encoder outputs to produce future trajectories:

(7) $$\hat{Y}=D_{\mathrm{Trans}}\left(q,E\right)=\left[{\hat{y}}_{\left(T_{\mathrm{obs}}+1\right)},\ldots ,{\hat{y}}_{T_{\mathrm{pred}}}\right].$$

These outputs correspond to the predicted future positions aligned with the fused behavioral and scene context.

Overall, this context-augmented prediction module enhances trajectory forecasting by combining temporal motion trends with semantic environmental understanding.

### 3.4. Risk-Aware Decision Reasoning

This module aims to identify high-risk objects in the driving scene, interpret the contextual factors contributing to those risks, and recommend appropriate vehicle responses through natural language reasoning. To achieve this, it relies on two inputs: the multimodal scene embedding Z generated from the cross-attention fusion of visual and textual cues, and a task prompt *P* designed for chain-of-thought reasoning.

Given these inputs, the MLLM processes Z together with the instruction prompt *P* and generates step-by-step explanations that: (1) pinpoint the most critical risky object in the given scene; (2) provide detailed explanations of why this object poses a risk; and (3) recommend appropriate vehicle responses.

To support this reasoning process, we adopt DeepSeek-R1-Distill-QWEN-7B [54] as the backbone MLLM. This model offers a favorable balance between efficiency and expressiveness, making it suitable for real-time autonomous driving scenarios. Crucially, it incorporates a CoT reasoning mechanism, which allows the model to break down complex driving situations into a series of intermediate reasoning steps. Rather than relying on a single forward pass to generate output, the model iteratively considers behavioral cues (e.g., object speed, trajectory curvature), spatial layouts (e.g., occlusions, road boundaries), and semantic context to arrive at logically consistent and context-aware conclusions.

Furthermore, DeepSeek-R1-Distill-QWEN-7B benefits from reinforcement learning-based alignment during pretraining. This optimization strategy improves the reliability, safety, and consistency of the model’s outputs, which is particularly valuable for safety-critical tasks such as risk interpretation and decision guidance in autonomous driving. By incorporating human preference modeling into the training loop, the model is encouraged to prioritize responses that are not only informative, but also aligned with human-like reasoning patterns.

## 4. Results

### 4.1. Experimental Details

#### 4.1.1. Dataset

The experiments were conducted using the Rank2Tell dataset [22], consisting of 116 video clips (an average of 20 s per clip) recorded from vehicle-mounted cameras positioned at the left, center, and right viewpoints. Although the dataset size is moderate, it provides diverse urban scenarios, including pedestrian crossings, lane blockages, and intersection conflicts, offering sufficient contextual diversity for evaluating both the trajectory prediction module and the reasoning component. Given the scope of this study, the dataset provides sufficient diversity to evaluate the performance of the proposed trajectory prediction pipeline as well as the behavior of the integrated reasoning module within the DSC-LLM framework.

#### 4.1.2. Training Setup

The proposed framework was trained to forecast object positions over a 5-s horizon ($T_{\mathrm{pred}}=5$) based on 5-s observation ($T_{\mathrm{obs}}=5$) windows. The model operates non-autoregressively, predicting all future positions simultaneously. Importantly, the weighting coefficient of 0.1 is applied to the MSE term, reflecting our emphasis on ensuring that the trajectory prediction remains stable and sufficiently influential within the multi-objective optimization. This design enables the model to prioritize accurate future coordinate estimation while still benefiting from auxiliary learning signals. The dataset was divided into training and test sets in an 8:2 ratio. The MLLM component was deactivated during training and activated only at inference, while the pretrained vision encoder was employed in both training and inference with its parameters kept frozen.

#### 4.1.3. MLLM Configuration for Risk-Aware Reasoning

For the inference task of risk-aware decision reasoning, we employed publicly available checkpoints of DeepSeek-R1-Distill-Qwen-7B, Mistral-7B-Instruct-v0.3, and LLaMA-3.1-8B from Hugging Face. To ensure reproducibility, we adopted the default inference configurations provided in the respective model cards. Specifically, the maximum context length was set to 4096 tokens for Qwen, 8192 tokens for Mistral, and 8192 tokens for LLaMA-3.1, respectively. Sampling hyperparameters were set to a temperature of 0.7, top-p of 0.9, and top-k between 40–50 depending on the model variant. Furthermore, to mitigate randomness in generation and secure consistent comparisons, random seeds were fixed at 42.

### 4.2. Evaluation Metrics

Trajectory prediction performance was evaluated using three standard metrics: average displacement error (ADE) [13], final displacement error (FDE) [13], and root mean squared error (RMSE). ADE measures the average Euclidean distance between predicted and ground-truth trajectories, while FDE focuses only on the final predicted position. To ensure robustness under the limited dataset size, we conducted evaluation with five random seeds (0, 42, 123, 2025, 777) and reported averaged results, denoted as ADE-avg, FDE-avg, and RMSE-avg. Given the current limitations of the Rank2Tell dataset, which does not yet provide full coverage of scene-level risk annotations and explicit rankings, conventional quantitative metrics for decision reasoning are difficult to apply. Therefore, qualitative evaluation was additionally performed. To ensure consistency and minimize subjectivity, we explicitly defined and applied three criteria throughout all comparisons: (1) accuracy of scene understanding, (2) contextual plausibility, (3) relevance and practicality of recommended actions.

### 4.3. Quantitative Evaluation for Trajectory Prediction

#### 4.3.1. Baseline Experiment

Since no prior work has performed trajectory prediction directly on the Rank2Tell dataset, we established our own set of baselines. Specifically, TCN and LSTM were chosen as representative temporal modeling architectures widely adopted in trajectory prediction, while the Transformer was employed as a strong sequence modeling backbone to validate the effectiveness of our proposed TME architecture. According to the results summarized in Table 1, the proposed model consistently outperforms three baseline architectures, namely temporal convolutional network (TCN), LSTM, and a standard Transformer, across all evaluation metrics. All models were trained and evaluated under identical conditions to ensure a fair comparison. The proposed model achieved an ADE-avg of 12.272, FDE-avg of 14.889, and RMSE-avg of 9.752. Compared to the transformer (ADE-avg: 17.598, FDE-avg: 20.825, RMSE-avg: 13.426), the proposed model achieves relative improvements of 30.3%, 28.5%, and 27.4%. When compared with the TCN (ADE-avg: 18.429, FDE-avg: 24.129, RMSE-avg: 14.203), the gains become more pronounced, reaching 33.4%, 38.3%, and 31.3%. Furthermore, even against the LSTM model-which is inherently designed to capture temporal dependencies-the proposed method yields additional reductions of 6.6% in ADE-avg, 6.2% in FDE-avg, and 5.2% in RMSE.

These results substantiate the effectiveness of incorporating temporally accumulated context into the prediction framework. The consistent outperformance of the proposed model across all baseline architectures highlights its enhanced capability to represent complex motion patterns in dynamic environments. Furthermore, the LSTM baseline outperforms both the Transformer and TCN, suggesting that the ability to capture long-term temporal dependencies plays a critical role in trajectory forecasting. This observation further supports the design rationale of our approach, as explicitly encoding temporally structured context improves the model’s ability to capture motion dynamics and generate more accurate and stable trajectory predictions.

#### 4.3.2. Ablation Study

An ablation study was conducted to quantitatively evaluate the contribution of each component in the DSC representation, including visual features (image), dynamic status, velocity, and yaw angle. Table 2 summarizes the results, showing that the model utilizing all features achieved the highest performance. While the removal of visual features resulted in the most noticeable degradation, other elements such as velocity, dynamic status, and yaw angle also contributed complementary information. In particular, these temporal features enhanced the predictive capability when combined with spatial cues from images. These results indicate that the purpose of this ablation study is not to remove entire modules, but to analyze how each feature contributes to the DSC representation, underscoring the necessity of jointly integrating spatial and temporal features for accurate trajectory prediction. It should be noted that this ablation is conducted on input features, aiming to investigate how different configurations of the DSC representation affect trajectory prediction performance.

### 4.4. Qualitative Evaluation for Risk-Aware Decision Reasoning

This section evaluates how reinforcement learning training and CoT optimization influence interpretability in risk-aware decision reasoning. To this end, we conducted a comparative qualitative analysis using three representative models with different training pipelines. LLaMA3.1-8B [55] relies on autoregressive next-token pretraining only, without any additional fine-tuning. Mistral-7B-Instruct-v0.3 [56] extends the same autoregressive pretraining with supervised instruction fine-tuning on publicly available datasets, but does not include reinforcement learning. DeepSeek-R1-Distill-Qwen-7B inherits pretrained and instruction-tuned weights from Qwen-7B and further incorporates a reinforcement learning objective into its training framework. Importantly, to ensure fairness, CoT prompting was applied not only to DeepSeek but also to LLaMA and Mistral, allowing all models to benefit from structured reasoning when generating responses. All models received identical multimodal inputs consisting of visual features and language prompts, and were guided to identify potential risks, explain their causes, and recommend appropriate driving responses.

We qualitatively evaluate these models across three representative driving situations: (1) a pedestrian crossing scenario, (2) a right-side vehicle intrusion scenario, and (3) a no-risk scenario. These cases collectively cover the primary categories of real-world hazards—vulnerable road users, interacting vehicles, and absence of actionable risk—and allow us to assess each model’s ability to reason about risk severity, causal factors, and appropriate driving responses.

**Figure 2:**
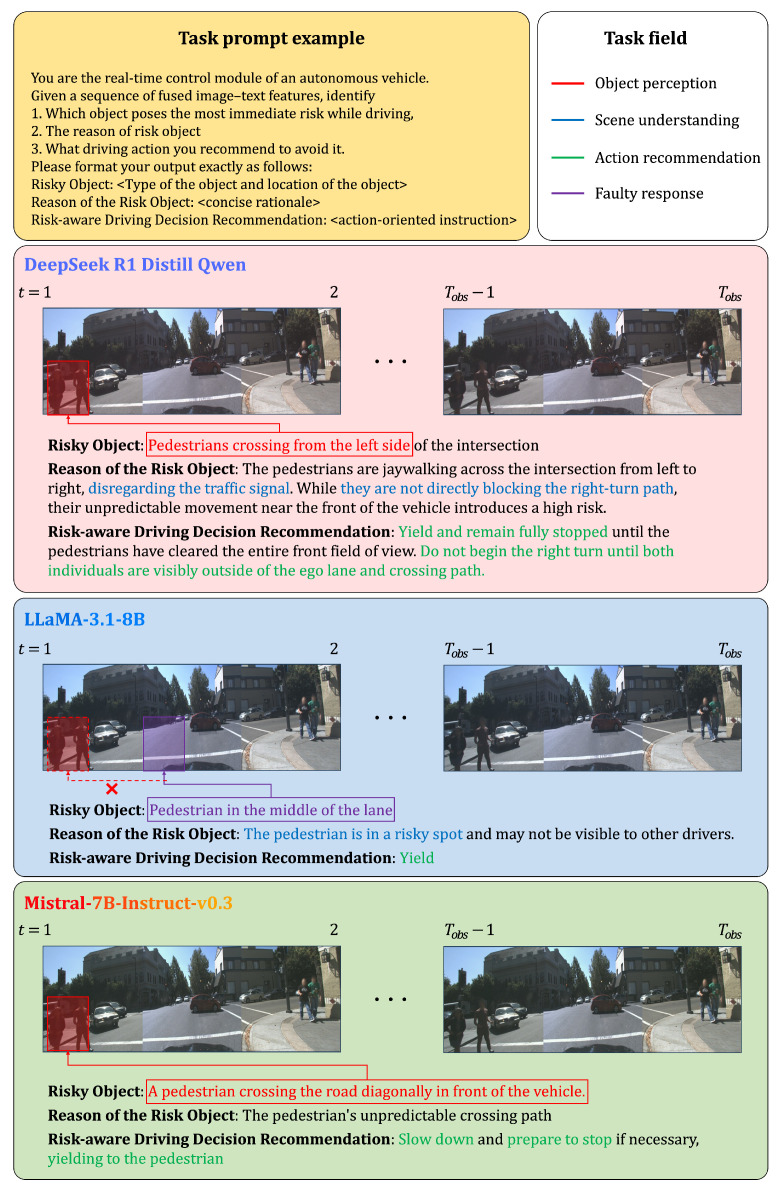
Comparison of risk-aware decision reasoning in a pedestrian-crossing scenario.

As the chosen model in our proposed framework, DeepSeek-R1-Distill-Qwen-7B demonstrated superior interpretability by generating responses that (1) accurately identify the correct risky object (or recognize the absence of risk), (2) provide contextually grounded explanations, and (3) recommend actionable and appropriate driving behaviors. For example, in the pedestrian-crossing case (Figure 2), DeepSeek correctly pinpointed the crossing pedestrians as the primary hazard and explained its decision using detailed contextual cues such as jaywalking behavior, direction of movement, and proximity to the ego vehicle’s intended turning path. Its recommendation to remain fully stopped until all pedestrians cleared the roadway reflects high consistency between perception and action.

**Figure 3:**
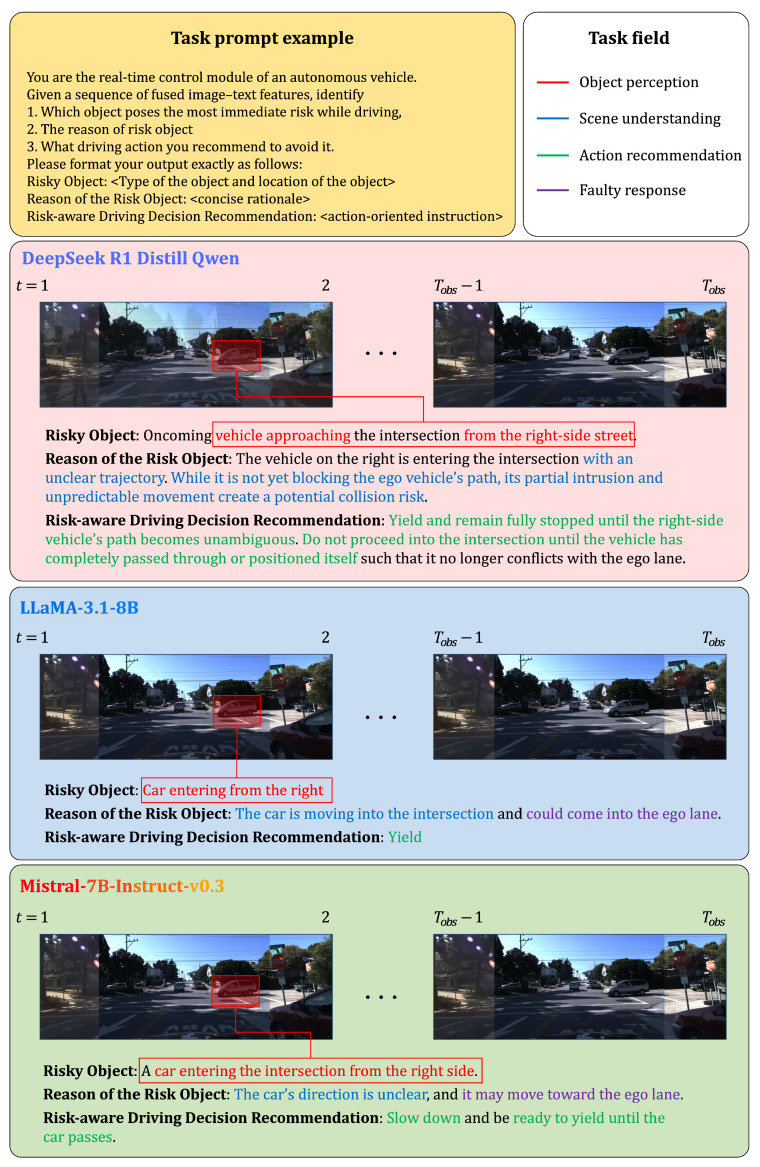
Comparison of risk-aware decision reasoning in an oncoming-vehicle interaction scenario.

In the vehicle intruding from the right-side street (Figure 3), DeepSeek again provided the most coherent interpretation by describing the vehicle’s partial intrusion and unclear trajectory as the source of risk, and recommending a defensive yielding maneuver until the oncoming vehicle’s motion became unambiguous. This demonstrates its ability to reason about dynamic interactions rather than only recognizing object presence.

**Figure 4:**
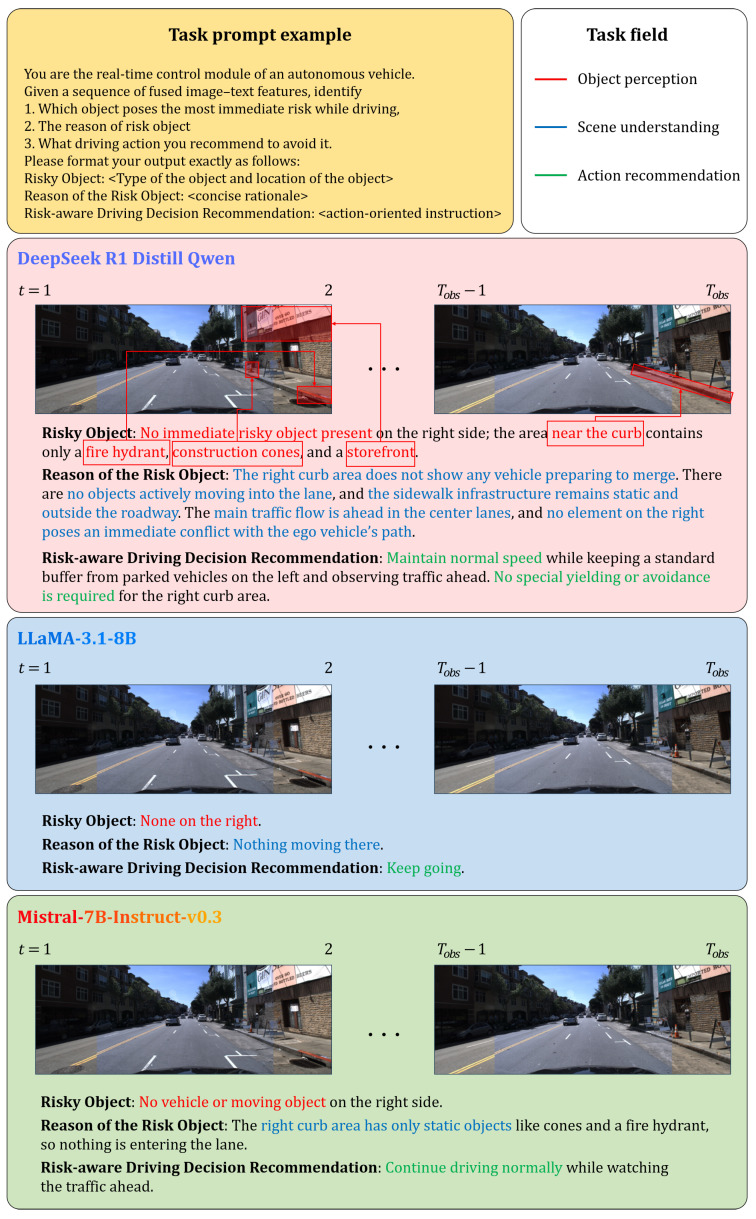
Comparison of risk-aware reasoning under a no-immediate-risk scenario.

In the no-immediate-risk scenario (Figure 4), DeepSeek successfully distinguished static roadside elements (e.g., fire hydrant, cones, storefronts) from actual moving hazards, explaining why none of these objects posed a collision risk. Its recommendation to maintain normal driving speed shows well-calibrated negative-risk judgment, avoiding unnecessary caution while still acknowledging scene context.

Although LLaMA3.1-8B and Mistral-7B-Instruct-v0.3 also employed CoT prompting and were able to identify risk objects in many cases, their reasoning was often shallow, fragmented, or overly generic. For example, LLaMA frequently misinterpreted spatial relationships or produced under-specified rationales, while Mistral tended to overgeneralize hazards or provide vague recommendations lacking situational precision. In contrast, DeepSeek-R1-Distill-Qwen-7B generated structured and interpretable outputs with greater consistency, a result attributed to the combination of reinforcement learning and CoT optimization.

These findings demonstrate that reinforcement learning, in addition to structured multimodal representation and CoT-based alignment in DeepSeek-R1-Distill-Qwen-7B, further strengthens the robustness and interpretability of decision reasoning in autonomous driving systems. By applying CoT prompting consistently across all compared models, our evaluation isolates the additional benefits provided by reinforcement learning and multimodal integration in the proposed framework.

### 4.5. Runtime Efficiency Analysis

To further validate the real-time applicability of our proposed framework, we measured the computational complexity and inference efficiency of the trajectory prediction module, which is the core component for ensuring safe motion planning. On an NVIDIA GeForce RTX 4090 GPU (NVIDIA, Santa Clara, CA, USA), the module requires approximately 6.13 M trainable parameters and 60.97 M FLOPs per inference, achieving an average latency of 4.12 ms per sample (about 242 FPS). These results confirm that trajectory prediction can be performed in real time, which is critical for ensuring timely and safe control in autonomous driving systems. In contrast, the risk-aware decision reasoning component mainly serves interpretability and high-level risk assessment, and therefore does not demand the same strict real-time guarantees as the trajectory prediction module. Ensuring the real-time performance of trajectory prediction is thus sufficient for safe deployment, while decision reasoning complements the framework by providing additional interpretability and safety awareness.

### 4.6. Discussion

This study proposes an integrated framework that couples scene-aware trajectory prediction with MLLM–based risk reasoning. The experimental results demonstrate that the proposed architecture is able to generate accurate motion forecasts while producing coherent, context-grounded explanations for potential hazards in urban driving scenarios. By unifying these two components within a single pipeline, the framework offers an interpretable decision-support mechanism that complements conventional trajectory-only predictors.

Despite these strengths, several limitations remain. First, while standard trajectory metrics such as ADE and RMSE effectively quantify spatial prediction accuracy, they do not directly evaluate the interpretability or reasoning fidelity of the MLLM component. The current study qualitatively validates that the reasoning module identifies hazardous agents and provides logically consistent justifications; however, it does not measure whether such reasoning correlates with changes in predicted motion, aligns with human-annotated causal factors, or results in reduced collision likelihood. Establishing these forms of correlation requires dedicated evaluation protocols and large-scale human-annotated datasets—resources that fall outside the scope of the Rank2Tell benchmark and the present study.

Second, the reasoning module is activated only at inference time, meaning that its outputs do not directly influence the trajectory prediction network during training. Although this design allows the framework to remain modular and lightweight, it also limits the system’s ability to learn joint causal relationships between perceived risks and motion outcomes. Future work may explore reinforcement learning or differentiable surrogate objectives to more tightly couple reasoning signals with motion forecasting behaviors.

Third, the concept of “risk” in this study is not defined through a formal traffic-engineering taxonomy. Instead, the model infers risk implicitly from multimodal cues—visual semantics, contextual motion information, and language-grounded descriptions. This design reflects the inherent limitations of the Rank2Tell dataset, which does not provide ground-truth risk annotations or causal labels. As a result, the proposed reasoning module serves as an interpretability mechanism that exposes contextual factors influencing trajectory predictions, rather than a safety-critical risk assessment system. To partially address this limitation, the qualitative evaluation was expanded to include three representative scenario categories (vehicle–pedestrian interactions, vehicle–vehicle interactions, and no-risk scenes), enabling a more comprehensive examination of how the model identifies and explains potential hazards across diverse driving situations.

Finally, the current experiments are conducted on relatively short video clips and a finite set of urban scenarios. While these are sufficient to demonstrate architectural feasibility and consistent reasoning behavior, broader validation across more diverse environments and adversarial conditions would further substantiate the framework’s generalizability.

In summary, this work provides evidence that combining MLLM-based reasoning with trajectory prediction is feasible and yields interpretable decision-support outputs. Future research will focus on developing quantitative metrics for reasoning fidelity, constructing evaluation protocols that measure causal alignment between reasoning and motion predictions, defining risk with greater traffic-engineering rigor, and extending the system to larger-scale and more complex driving datasets.

## 5. Conclusions

This study proposed DSC-LLM, a framework that integrates temporal behavioral features and visual context to support both trajectory prediction and interpretable risk reasoning. The framework achieved the best performance among all baselines on the Rank2Tell dataset (ADE-avg 12.272, FDE-avg 14.889, RMSE-avg 9.752), with five-seed evaluation and ablation analyses confirming that each DSC component contributes complementary information.

In parallel, the reasoning module serves as an interpretable layer that exposes the contextual factors underlying each predicted trajectory. Its effectiveness was validated through structured qualitative analysis across three representative scenarios—pedestrian crossing, right-side vehicle intrusion, and no-risk cases. DeepSeek-R1-Distill-Qwen-7B consistently produced the most accurate risk identification and context-aligned explanations, confirming that the DSC representation provides reliable evidence for interpretable reasoning.

Overall, the results show that DSC-LLM provides a coherent pipeline in which trajectory prediction serves as the primary objective and multimodal reasoning complements it by revealing the contextual factors behind each motion estimate. This integration offers practical value by pairing future trajectory forecasts with scene-grounded explanations that support safer decision-making and post hoc analysis. These conclusions are supported by quantitative improvements over strong baselines and consistent qualitative validation across multiple driving scenarios, confirming the reliability of the proposed framework.

In future work, we plan to expand the dataset and conduct larger-scale evaluations to further validate the generalization and stability of the proposed DSC-LLM framework. Additionally, we aim to explore tighter coupling between prediction and reasoning modules, enabling more coherent safe-path planning that reflects both motion forecasts and context-aware risk assessments.

## Figures and Tables

**Table 1:** Result of performance evaluation.

Model	ADE-avg	FDE-avg	RMSE-avg
TCN	18.429 ± 0.535	24.129 ± 0.647	14.203 ± 0.339
Transformer	17.598 ± 2.123	20.825 ± 2.765	13.426 ± 1.507
LSTM	13.137 ± 1.256	15.865 ± 1.304	10.282 ± 0.846
**Ours**	**12.272** ± **1.006**	**14.889** ± **1.054**	**9.752** ± **0.757**

**Table 2:** Ablation study on the components of DSC features for trajectory prediction.

Image	Dynamic/Static	Velocity	Yaw	ADE	FDE	RMSE
✓	✓	✓	–	11.570	14.079	9.193
✓	✓	–	✓	13.874	16.481	10.571
✓	–	✓	✓	12.055	14.791	9.517
–	✓	✓	✓	14.622	17.351	11.317
✓	✓	✓	✓	**10.972**	**13.701**	**8.782**

Note: The check mark (✓) indicates inclusion of the feature, while “–” indicates exclusion.

## Data Availability

The datasets used in this study are described in the manuscript. The Rank2Tell dataset is publicly accessible for non-commercial research use at https://usa.honda-ri.com/rank2tell (accessed on 15 January 2025). All experiments were conducted in accordance with the dataset’s usage policy. Processed data generated during the study are not publicly released due to dataset redistribution restrictions, but they are available from the corresponding author upon reasonable request.

## Abbreviations

The following abbreviations are used in this manuscript:

ADE	Average Displacement Error
Bi-LSTM	Bidirectional Long Short-Term Memory
CLIP	Contrastive Language-Image Pretraining
CoT	Chain-of-Thought
DSC	Driving Scene Context
FDE	Final Displacement Error
GNNs	Graph Neural Networks
LLMs	Large Language Models
LoRA	Low-Rank Adaptation
LTSF-NLinear	Long-Term Time Series Forecasting Normalization Linear
MLLM	Multimodal Large Language Model
MLP	Multi-Layer Perceptron
MSE	Mean Squared Error
RMSE	Root Mean Squared Error
TCN	Temporal Convolutional Network
TME	Temporal Multimodal Embedding
VLMs	Vision-Language Models
